# Aging, Disability, and Informal Caregivers: A Cross-sectional Study in Portugal

**DOI:** 10.3389/fmed.2017.00255

**Published:** 2018-01-16

**Authors:** Maria Ana Pego, Carla Nunes

**Affiliations:** ^1^Unidade de Investigação e Desenvolvimento em Enfermagem, Escola Superior de Enfermagem de Lisboa, Lisbon, Portugal; ^2^Centro de Investigação em Saúde Pública, Escola Nacional de Saúde Pública, Universidade Nova de Lisboa, Lisbon, Portugal; ^3^Escola Nacional de Saúde Pública, Universidade Nova de Lisboa, Lisbon, Portugal

**Keywords:** informal care, dependent elderly, epidemiology, aging, Portugal

## Abstract

**Objectives:**

Aging is pushing states to rethink long-term care policies in several dimensions. This study aims to characterize the reality of dependent older people regarding their demographic and health characteristics, to describe their informal carers and understand the availability of informal care.

**Methods:**

A cross-sectional study was developed in Portugal in 2013. Descriptive statistical analyses and binary logistic analysis were conducted.

**Results:**

Results show that the informal long-term care sector is primarily aimed at older people with severe limitations in their activities of daily living and at the chronically ill, particularly older women. Additionally, 39.5% of dependent older persons do not have informal care and only receive informal aid in cases of extreme need.

**Discussion:**

Results show a critical situation for both social groups (older persons and caregivers) and the prospect of an alarming situation in the near future (aging and reduced availability of informal caregivers) unless a new approach for long-term care is developed.

## Introduction

Improvements in living conditions and the quality of health services have led to a significant increase in the proportion of older people in the population, a trend that is expected to continue over the next decades. Public health authorities face the challenge of promoting the reform of health policies that are aimed at older people, due to the progressive loss of functionality and the consequent increase in dependence for their activities of daily living (ADL), leading the older person to an increased need for long-term care. These personal care components are often provided in combination with basic medical services (medication, health monitoring, prevention, rehabilitation, or palliative care services) and, sometimes, with lower level help, namely those related with Instrumental Activities of Daily Living (IADL), such as meals, shopping, and housework (1).

At first glance, one realizes that demographic, social, and economic changes are “pushing” states to reassess long-term care policies, mainly due to the restructuring of the family leading to a reduction in the availability of informal caregivers. The atomization of families, the greater volatility of marital relations, the reduction in the average size of families, declining birth rates, and the increasing participation of women in the labor market have substantially altered family structures and functioning (2).

Informal care is generally defined as the unpaid care provided to older and dependent people by a person with whom they have a social relationship, such as a spouse, parent, child, other relative, neighbor, friend, or other non-kin (3). In Europe, there is a multiplicity of forms of organization for long-term care, in which the North tends to have a greater provision of public services and formal care and the South favors the informal care sector provided by the family, due to the limited availability of formal resources (namely, systems of social protection for older people) and a “familiar” culture (4–10). For example, a significant proportion of long-term care is provided in this way in Spain (in 2008, 78% of care was provided by informal caregivers) and in France (in 2008, this amounted to 37%), according to Costa-Font and Courbage (11). Portugal shares the main characteristics of other Southern European countries, i.e., the family (in particular women) is the main provider of care, but differs in one key aspect: it has a high female participation rate in the labor market (9, 10, 12–14).

In a context of global aging and significant changes in family structures, it is necessary to comprehend the reality of informal care in order to understand its relevance to the care of the aging population. This study specifically contributes to the knowledge of the informal sector of care and aims to (i) describe the national reality of the dependent older person in Portugal regarding demographic, residential, and health characteristics; (ii) describe the informal carers, in terms of kinship, frequency of care, and geographical proximity between carer and older person; (iii) understand the availability of informal care. This article focuses on long-term care for the elderly in Portugal, framed in a context of significant changes in family structures and shifts in social solidarity networks. Thus, we argue that the current model of long-term care, based mainly on the informal sector, has not been able to keep up with the actual demands of the elderly in Portugal. Hence, we expect to find a significant part of the geriatric population in need of care. We also expect to find a relationship between the severity of the limitations perceived by older persons and the availability of an informal carer, i.e., only the most dependent and elderly have access to an informal caregiver.

## Materials and Methods

### Data Source

This study is cross-sectional, observational, and analytical. Data are obtained from the fourth wave of the Survey of Health, Aging and Retirement in Europe (SHARE) (15), the first one that included Portugal. SHARE is the first longitudinal and cross-national research project collecting data on aging and access to the informal care sector.

In each country, data collection is based on probability household samples (drawn either by simple random selection or multistage random selection) where all people aged above 50 years and their partners were interviewed using Computer Assisted Personal Interviews. Although the sampling frame includes people living in institutions, the SHARE base sample excludes these individuals. For the Portuguese SHARE sample, the target population was defined as all Portuguese-speaking residents, born before 1960 and their spouses/partners. For each country, the sample size originally defined was 2,000 individuals. In Portugal response rate of 60%, was expected, with an expected proportion of non-sampling units of 10%. Analysis of the width and magnitude of the 95% confidence interval (95% CI) were used in order to evaluate the uncertainty of estimates. The SHARE study (fourth wave) was subject to several ethics reviews: The Ethics Committee of the University of Mannheim, Germany, Ethics Council of the Max Planck Society and by national ethics committees or institutional review boards whenever this was required. This study was conducted in full accordance with the World Medical Association Declaration of Helsinki. Written consents from all participants involved in this study were obtained.

### Study Sample

For the purpose of this study two additional restrictions were applied: (1) 65 years or older and (2) functional dependence [limited in at least one ADL or one Instrumental Activity of Daily Living (IADL)]. In order to restrict the samples according to the functional dependence of the older person, we used the Katz Index (16) to characterize the ADL, and the Lawton and Brody Scale (17) to characterize the IADL.

Table 1 presents the variables used in the analysis to characterize dependent older people and informal caregivers.

**Table 1:** Variables used in the analysis to characterize dependent old person and informal caregivers.

Variables	Categories
**Dependent old person**	
Sex	Male; female
Age	65–69; 70–74; 75–79; 80–84; 85–99
No. of children	0; 1; 2; 3^a^; 4^a^; 5^a^; 6 or more^a^
Marital status	Married or registered partnership, living with spouse; married, separated from spouse^a^; never married^a^; divorced^a^; widowed^a^
Type of building	Farm house; 1 or 2 family house; building with three or more flats
Household area	Major city^a^; small city^a^; village^a^; rural area
Educational level	First level (4 years); second level^a^ (6 years); third level^a^ (9 years); secondary school; higher education; no education
Limitation^b^	Severely limited; limited, but not severely^a^; not limited
Chronic illness	^a^Yes; no

**Informal caregivers**	
Kinship	Daughters and sons (named as “children”); brother or sister; spouse; son-in-law or daughter-in-law; neighbor; other relative; housekeeper; grandchildren; friend
Gender	Male; female
Geo. proximity	Live in the same house; live in the same building; live less than 1 km away; between 1 and 5 km away; between 5 and 25 km away; between 25 and 100 km away; more than 100and away
Frequency of care	Daily care; weekly care; monthly care; less-frequent care

*^a^Categories merged in multivariate analyses, especially due to small frequencies*.

*^b^Perceived limitations on ADL––derives from the following question: “For the past six months at least, to what extent have you been limited because of a health problem in activities people usually do?”*

### Statistical Analysis

Descriptive statistical analyses were conducted to characterize the dependent elderly population and the informal caregivers, considering the variables presented in Table 1. Thereafter, binary and multivariate logistic analyses (using both methods: enter and forward LR: likelihood ratio) were used to characterize the availability of informal care (Model 1), being cared for by someone within the household, named here “inside care” (Model 2), being cared for by someone outside the household, named here “outside care” (Model 3). Considering the above variables (Table 1), crude, adjusted (by sex and age and by all significant variables), and corresponding confidence intervals were presented. We used SPSS statistical software with a 0.05% significance level. All models were tested and validated, namely through Hosmer and Lesmeshow’s goodness-of-fit test and analyses of residuals.

## Results

The findings from the descriptive statistics are presented in Table 2, globally (276) and stratified by type of help: without informal care (109), with inside care (89), and with outside care (78). The categories that best define each profile are in bold.

**Table 2:** Descriptive statistics.

Categories	All, *n* (%)	Without informal care, *n* (%)	Outside care, *n* (%)	Inside care, *n* (%)
**Gender**				
Male	91 (33.0)	43 (39.4)	28 (31.5)	20 (25.6)
Female	**185 (67.0)**	**66 (60.6)**	**61 (68.5)**	**58 (74.4)**
Total	276 (100)	109 (39.5)	89 (32.2)	78 (28.3)

**Age**				
65–69	**68 (25.9)**	**34 (31.2)**	19 (22.6)	15 (21.4)
70–74	48 (18.3)	32 (29.4)	8 (9.5)	8 (11.4)
75–79	67 (25.5)	28 (25.7)	17 (20.2)	**22 (31.4)**
80–84	53 (20.2)	12 (11.0)	**26 (30.9)**	15 (21.4)
85–99	27 (10.3)	3 (2.8)	14 (16.7)	10 (14.3)
Total	263 (100)	109 (41.4)	84 (31.9)	70 (26.6)

**Number of children**				
0	18 (7.7)	4 (3.7)	8 (16.7)	6 (7.8)
1	56 (23.9)	25 (22.9)	10 (20.8)	21 (27.3)
2	**80 (34.2)**	**42 (38.5)**	**16 (33.3)**	**22 (28.6)**
3	34 (14.5)	19 (17.4)	3 (6.3)	12 (15.6)
4	15 (6.4)	10 (9.2)	1 (2.1)	4 (5.2)
5	10 (4.3)	3 (2.8)	3 (6.3)	4 (5.2)
6 or more children	21 (9.0)	6 (5.5)	7 (14.6)	8 (10.4)
Total	234 (100)	109 (46.5)	48 (20.5)	77 (32.9)

**Marital status**				
Married^a^	**175 (63.4)**	**84 (77.1)**	**66 (74.2)**	25 (32.1)
Married, separated from spouse	2 (0.7)	0 (0)	0 (0)	2 (2.6)
Never married	8 (2.9)	2 (1.8)	2 (2.2)	4 (5.1)
Divorced	8 (2.9)	3 (2.8)	2 (2.2)	3 (3.8)
Widowed	83 (30.1)	20 (18.3)	19 (21.3)	**44 (56.4)**
Total	276 (100)	109 (39.5)	89 (32.2)	**78 (28.3)**

**Type of building**				
Farm house	12 (5.8)	6 (6.5)	1 (2.3)	5 (7)
1 or 2 family house	**104 (50.0)**	**51 (54.8)**	21 (47.7)	32 (45.1)
Building with 3 or more flats	92 (44.2)	36 (38.7)	**22 (50)**	**34 (47.9)**
Total	208 (100)	93 (44.7)	**44 (21.2)**	**71 (34.1)**

**Household area**				
Major city	**89 (42.8)**	**36 (38.7)**	**19 (43.2)**	**34 (47.9)**
Small city	33 (15.9)	16 (17.2)	8 (18.2)	9 (12.7)
Village	24 (11.5)	11 (11.8)	3 (6.8)	10 (14.1)
Rural area	62 (29.8)	30 (32.3)	14 (31.8)	18 (25.4)
Total	208 (100)	93 (44.7)	44 (21.2)	71 (34.1)

**Educational level**				
First level (4 years)	**147 (53.8)**	**62 (56.9)**	**47 (53.4)**	**38 (50)**
Second level^a^ (2 years)	19 (7.0)	8 (7.3)	5 (5.7)	6 (7.9)
Third level^a^ (3 years)	15 (5.5)	4 (3.7)	8 (9.1)	3 (3.9)
Secondary school	16 (5.9)	5 (4.6)	5 (5.7)	6 (7.9)
Higher education	14 (5.1)	8 (7.3)	4 (4.5)	2 (2.6)
No education	62 (22.7)	22 (20.2)	19 (21.6)	21 (27.6)
Total	273 (100)	109 (39.9)	88 (32.2)	76 (27.8)

**Perceived limitations in ADL**				
Severely limited	**144 (52.2)**	28 (25.7)	**67 (75.3)**	**49 (62.8)**
Limited, but not severely	98 (35.5)	**60 (55.0)**	17 (19.1)	21 (26.9)
Not limited	34 (12.3)	21 (19.3)	5 (5.6)	8 (10.3)
Total	276 (100)	109 (39.5)	89 (32.2)	78 (28.3)

**Chronic illness**				
Yes	**216 (78.3)**	**71 (65.1)**	**77 (86.5)**	**68 (87.2)**
No	60 (21.7)	38 (34.9)	12 (13.5)	10 (12.8)
Total	276 (100)	109 (39.5)	89 (32.2)	78 (28.3)

*^a^Or registered partnership, living with spouse*.

Table 2 shows that women were a majority in all situations, which is as expected because the sample includes more women than men. Regarding age, it is also likely that the most elderly tend to have more informal inside care. Regarding marital status, the most striking aspect is the fact that, among those who receive outside care, the majority are widowed (56.4%). Regarding the number of children, it appears that those who receive outside care differ from the rest. Indeed, they are characterized by having three or more children. Regarding the type of accommodation there is a clear distinction between those who do not have an informal carer and live primarily in a family home (54.8%), and those who have an informal carer and live primarily in buildings with more than three flats. Table 2 shows that among those who have no care and those who are less dependent in ADL form the majority (55.0%).

The percentage of chronically ill is considerably lower among those who do not have informal care. However, it should be noted that even those who do not receive informal care have a very high percentage of chronically ill (65.1%). Inside caregivers are mainly spouses (55.5%) and adult children (24.2%), and the remaining caregivers are neighbors, housekeepers, friends, and other relatives. Of the spouses 56.3% are male and 43.6% female, and 71.4% of the adult children caregivers are female and 62.5% of the remaining caregivers are female. Table 3 shows that the primary outside care providers are adult children (38.6%), followed by neighbors (15.9%), friends (8.4%), housekeepers (7.5%), and other relatives (27.7%).

**Table 3:** Characterization of the informal caregivers––outside care.

	Daily, *n* (%)	Weekly, *n* (%)	Monthly, *n* (%)	Less freq., *n* (%)	All, *n* (%)
**Kinship**					
Spouse	1 (1.89)	1 (2.70)	0 (0)	0 (0)	2 (1.68)
Children	21 (39.62)	18 (48.65)	1 (14.29)	6 (27.27)	46 (38.66)
Brother or sister	3 (5.66)	3 (8.11)	0 (0)	2 (9.09)	8 (6.72)
Daughter-in-law or son-in-law	3 (5.66)	3 (8.11)	2 (28.57)	1 (4.55)	9 (7.56)
Neighbor	8 (15.09)	5 (13.51)	1 (14.29)	5 (22.73)	19 (15.97)
Housekeepers	6 (11.32)	2 (5.41)	0 (0)	1 (4.55)	9 (7.56)
Grandchildren	4 (7.55)	2 (5.41)	0 (0)	1 (4.55)	7 (5.88)
Friend	5 (9.43)	0 (0)	2 (28.57)	3 (13.64)	10 (8.40)
Other relative	2 (3.77)	3 (8.11)	1 (14.29)	3 (13.64)	9 (7.56)
Total	53 (100)	37 (100)	7 (100)	22 (100)	119(100)

**Gender**					
Male	13 (35.14)	10 (34.48)	2 (33.33)	9 (56.25)	
Female	24 (64.86)	19 (65.52)	4 (66.67)	7 (43.75)	
Total	37 (100)	29 (100)	6 (100)	16 (100)	

**Geographic proximity**					
In the same household	12 (37.5)	4 (13.79)	0 (0)	1 (10)	
In the same building	2 (6.25)	0 (0)	0 (0)	1 (10)	
Less than 1 km away	11 (34.38)	10 (34.48)	1 (25)	4 (40)	
Between 1 and 5 km away	2 (6.25)	1 (3.45)	0 (0)	1 (10)	
Between 5 and 25 km away	5 (15.63)	9 (31.03)	1 (25)	1 (10)	
Between 25 and 100 km away	0 (0)	5 (17.24)	2 (50)	1 (10)	
More than 100 km away	0 (0)	0 (0)	0 (0)	1 (10)	
Total	32 (100)	29 (100)	4 (100)	10 (100)	

The majority of daily care is delivered by adult children (39.6%), other relatives (24.5%, including spouses, brothers or sisters, grandchildren, daughters or sons-in-law), neighbors (15.1%), and housekeepers (11.3%). These caregivers are predominantly female (64.8%) and live less than a mile away (78.13%).

As for weekly care, this is performed mainly by adult children (48.6%), other relatives (32.4%), and neighbors (13.5%); the caregivers are predominantly female (65.5%) and most live less than a kilometer away (48.2%). As shown in Table 3, as the frequency of care decreases, the distance between the caregiver and the person cared for increases. To explain and model the availability of informal carers for older persons in need of care, binary logistic models were used (Model 1—all informal care, Model 2—inside care, and Model 3—outside care). OR and CI of statistically significant variables are presented in Table 4.

**Table 4:** Model 1, 2, and 3––results from the logistic regression analyses.

Model 1––availability of informal care			

Independent variables	Crude odds ratio (95% CI; *p*)	Adjusted odds ratio^a^ (95% CI; *p*)	Forward LR model,^b^ odds ratio(95% CI; *p*)
**Age**			
65–69 years^c^	–	–	–
70–74 years	0.498 (0.228:1.087; *p* = 0.08)	–	0.461 (0.149:1.433; *p* = *0.181*)
75–79 years	1.290 (0.644:2.585; *p* = *0.473*)	–	1.532 (0.576:4.078; *p* = *0.393*)
80–84 years	2.922 (1.289:6.623; *p* = *0.010*)	–	1.437 (0.475:4.345; *p* = *0.520*)
85–99 years	10.271 (2.847:37.050; *p* = 0.001)	–	13.173 (2.772:62.601; *p* = 0.001)

**Perceived limitations in the ADL**			
Little or no limitation^c^	–	–	–
Severely limited	6.429 (1.904:6.616; *p* = 0.001)	6.481 (3.555:11.813; *p* = 0.001)	8.840 (3.958:19.612; *p* = 0.001)

**Marital status**			
Married and living without a spouse^c^	–	–	–
Does not live with the spouse	2.581 (1.483:4.493; *p* = 0.001)	1.933 (1.025:3.646; *p* = 0.042)	5.100 (2.288:11.371; *p* = 0.001)

**Model 2––availability of informal care from someone inside the household**			

**Independent variables**	**Raw odds ratio (95% CI; *p*)**	**Adjusted odds ratio^a^ (95% CI; *p*)**	**Forward LR model,^b^ odds ratio (95% CI; *p*)**

**Age**			
68–69 years^c^	–	–	–
70–74 years	0.466 (0.189:1.148; *p* = *0.097*)	–	0.593 (0.128:2.742; *p* = 0.504)
75–79 years	1.156 (0.543:2.459; *p* = *0.707*)	–	1.704 (0.490:5.921; *p* = 0.402)
80–84 years	3.263 (1.544:6.895; *p* = *0.002*)	–	5.136 (1.471:17.930; *p* = 0.010)
85–99 years	6.200 (2.465:15.592; *p* = 0.001)	–	8.743 (2.236:34.180; *p* = 0.002)

**Perceived limitations in the ADL**			
Little or no limitation^c^	–	–	–
Severely limited	6.768 (3.843:11.919; *p* = 0.001)	6.414 (3.512:11.714; *p* = 0.001)	6.652 (2.236:34.180; *p* = 0.001)

**Model 3––availability of informal care from someone outside the household**			

**Independent variables**	**Raw odds ratio (95% CI; *p*)**	**Adjusted odds ratio^a^ (95% CI; *p*)**	**Forward LR model,^b^ odds ratio (95% CI; *p*)**

**Educational level**			
No education^c^	–	–	–
First level (4 years)	0.813 (0.424:1.559; *p* = 0.533)	0.896 (0.453:1.772; *p* = *0.7*52)	0.945 (0.446:2.003; *p* = 0.883)
Second (2 years) and third level (3 years)	0.779 (0.305:1.991; *p* = 0.602)	0.810 (0.292:2.251; *p* = *0.687*)	1.224 (0.39:3.846; *p* = 0.729)
Secondary education	1.633 (0.486:5.484; *p* = *0.428*)	1.543 (0.412:5.781; *p* = *0.520*)	9.436 (1.665:53.472; *p* = 0.011)
Higher education	0.272 (0.056:1.312; *p* = *0.105*)	0.239 (0.046:1.232; *p* = *0.087*)	0.253 (0.048:1.324; *p* = 0.104)

**Perceived limitations in the ADL**			
No limitation or little limitation^c^	–	–	–
Severely limited	2.923 (1.680:5.088; *p* = 0.001)	2.937 (1.635:5.274; *p* = 0.001)	2.316 (1.155:4.644; *p* = 0.018)

**Chronic illness**			
No^c^	–	–	–
Yes	3.370 (1.615:7.035; *p* = 0.001)	1.424 (0.745:2.722; *p* = *0.285*)	4.779 (1.668:13.691; *p* = 0.004)

**Marital status**			
Lives with the spouse^c^	–	–	–
Does not live with the spouse	3.165 (1.799:5.567; *p* = 0.001)	2.940 (1.492:5.795; *p* = 0.002)	2.772 (1.424:5.396; *p* = 0.003)

*^a^Adjusted to gender and age*.

*^b^Forward model LR, embodying all the variables under analysis*.

*^c^Reference class*.

Model 1 (G2 = 78,075; *p* < 0.001) has an explanatory capacity of 76%, with a sensitivity of 75.6% and a specificity of 76.3%. The corresponding values for Model 2 are G2 = 46,145 (*p* < 0.001), 78.8%, 71.4%, and 81.6% and for Model 3 are G2 = 41,008 (*p* < 0.001), 63.3%, 92.4%, and 50.3%.

Model 1 presents age, marital status, and limitations perceived in ADL as significant variables. Thus, it is observed that people aged 85–99 years have a 13.1 times higher probability of being informally cared for than those aged 65–69 years old. On the other hand, an older person living without a spouse is 5.1 times more likely to be informally cared for than an older person living with a spouse. It was also shown that older persons that perceive severe restrictions in their ADL have a probability 8.8 times higher of being informally cared for than those that perceive no limitation or little limitation in their ADL.

Model 2 identifies age and limitations perceived in ADL as significant variables: people aged 85–99 years have an 8.7 times greater likelihood of being informally cared for by someone within the household than people 65–69 years old. People aged 80–84 are 5.1 times more likely to be informally cared for than those aged 65–69 years old. Those that are severely limited are 6.6 times more likely to be cared for by an inside carer than those with little or no limitations in ADL.

Model 3 identifies education, marital status, perceived limitations in ADL, and the presence of chronic disease as significant variables. An older person with a secondary level of education is 9.4 times more likely to be cared for by an outside carer than an older person with no education. An older person living without a spouse is 2.8 times more likely to have outside care than those who live with their spouse. The results also show that an older person that perceives severe limitation in ADL is 2.3 times more likely to have outside care than those who perceive little or no limitation. Finally, older people with chronic disease have a 4.8 times higher probability of having outside care than those who are not chronically ill.

## Discussion

The majority of the informally cared for dependent elderly live with their main caregiver (53.3%). This result is expected, because older age groups are probably more dependent and in need of more frequent care and supervision in their daily activities. Similar studies (10) have shown that in Southern Europe, the main caregivers live inside the household. On the other hand, informal care is predominantly provided by family members (79.3%) (6, 18). Originally, it is the absence of a spouse that seems to influence the use of outside care, i.e., not having the possibility of being cared for by a spouse increases the probability of having a carer from outside the household.

Regarding outside care, it is mostly provided by adult children (38.6%), something that is also mentioned in the literature (10, 19). The main caregivers living outside the household are female. This is shown in several reports on Europe, especially Southern Europe (4, 9, 10, 20).

The results show that the main recipients are older women limited in their ADL, chronically ill, and of advanced age. The literature shows that women have a higher life expectancy; however, the quality of life after age 65 declines substantially due to limitations in ADL and chronic illness (21).

The main caregivers among coresident caregivers are spouses (55.5%). Furthermore, geographical distance is extremely relevant in determining the frequency of care. Caregivers that care occasionally live outside the household. This shows that there is a nuclearization of the family, supporting the argument made on changes in family structure (10, 22–24).

In addition to the characterization of informal caregivers, our study aims to highlight the limitations of the current model of care. A significant proportion (39.5%) of the dependent elderly do not receive any type of informal care. The literature highlights the limitations of the current model of long-term care imposed by changes in family structures (10, 22–24).

Regarding the availability of inside care (all care, inside or outside), one variable is consistently identified: the limitation perceived in ADL, and the higher the limitation is perceived, the greater the availability of informal carers.

People aged 80–99 years are more likely to have inside care, and older persons aged 85–89 years are more likely to have informal care. Other authors (4, 10, 19, 25) state that it is mainly the most elderly and therefore those who are more dependent who have more access to an informal caregiver. Thus, informal caregivers predominantly help the most vulnerable, dependent and older persons.

In the first and third models, the variable living without a spouse influences the availability of an informal caregiver; this means that in the absence of a spouse, older people are more likely to be informally cared for and cared for by an outside carer. Older persons residing without a spouse receive help from non-resident caregivers. Regarding the first model, it may be the fact that the spouses also have functional limitations which prevents them from providing care to their spouses. On the other hand, it turns out that being married and living with their spouse is not decisive for obtaining help from informal caregivers. This may be related to the similar percentage of married older persons and those living with a spouse among those without informal help. This does not mean that spouses do not provide care, but the marital status does not explain the availability of informal caregivers. Other studies (10, 23) reflect the same relationship between informal care and marital status, explaining that the care from a spouse is sometimes not regarded by the older person as care, and this influences the answers of the older person.

The third model has two explanatory variables that differ from the other two models: level of education and the presence of chronic disease. Older people with chronic illness are more likely to have informal care (4, 25). Older people with secondary and postsecondary education are more likely to be cared for by someone from outside, mainly by a housekeeper (7.56%). Similar data were found in a study on the Spanish situation in which users with secondary and postsecondary education are more likely to have paid care from paid domestic workers (26).

Finally, it appears that the number of children is not decisive for receiving help from informal caregivers. It is not the amount of available resources that determines whether or not there is access to long-term care. Daatland (27) states that it is the quality of the relationship between parents and children that favors the assumption of the caregiver role by the children and not the number of children.

In general the results of this study are aligned with the literature, reinforcing the internal and external validity of this study, and adding scientific evidence (namely through the magnitude of the results) to this study of this issue. Nevertheless, we would like to emphasize that these results should be carefully interpreted and generalized due to the fact that the sample under analysis is limited. The wide range of some confidence intervals reflects the uncertainty of these results. Additionally, data collection was based on self-report questionnaires (for instance, the perceived limitations in ADL and the presence of chronic illness variables) and participants may not have responded truthfully, either because they cannot remember (memory bias) or because they wish to present themselves in a more socially acceptable manner (social desirability bias). The absence of some relevant variables referred to in the literature (economic status, availability of formal care, health status of care giver, among others) also constitutes a limitation of this study.

## Conclusion

Based on this study, it appears that the informal long-term care sector in Portugal is primarily aimed at older people with severe limitations in their ADL and at the chronically ill, particularly older women. The absence of a spouse tends to mean that someone from outside the household responds to the need for care. Our study also shows that there are a significant proportion of dependent older persons without any kind of informal care (39.5%), which supports the argument that the current limitations of the long-term care model are imposed by changes in family structures.

Additionally, the main informal caregivers are family members and mostly women. However, the gender gap is not as clear when the primary caregiver is the spouse. Nonetheless, women represent 68% of the dependent elderly. Regarding adult children caregivers, the majority are female (71.4%). Among the caregivers who live with the older person, spouses are the main caregivers, in contrast to caregivers who live outside the household, where the majority are adult children and other relatives. It seems that there is a marked nuclearization of the family, supporting the argument in terms of the changes in family structure. On the other hand, the solidarity networks are mainly composed of family members. Regarding geographical distance, this is extremely relevant to the frequency of care, i.e., the closer the caregiver lives, the higher the frequency of care provided.

Taken as a whole, the current model of care, based on the informal long-term care sector, leaves out a significant proportion of dependent older persons and it seems that it is only in cases of extreme need that aid arises, i.e., only the most dependent and dependent older persons are helped by the informal care sector.

The results of this study show a critical situation for both social groups (older persons and caregivers) and an alarming prospect in the near future (a continuously aging society, a scarcity of young informal caregivers, the reduced availability of informal caregivers to care for the older person), something that is a global challenge, since many countries have the same characteristics. A new approach for long-term care is urgently required, a greater provision of public services and formal care is needed, especially at community and household level rather than institutionalization, allowing the elderly to live in their own context as long as is possible and promoting and safeguarding relationships and responsibilities with (and of) informal caregivers. Complementary studies on formal care are needed to understand the full context and to carefully evaluate different possible approaches (including elderly, caregivers and social/health services).

## Ethics Statement

The SHARE study (fourth wave) was subject to several ethics reviews: The Ethics Committee of the University of Mannheim, Germany, Ethics Council of the Max Planck Society and by national ethics committees or institutional review boards whenever this was required. This study was conducted in full accordance with the World Medical Association Declaration of Helsinki. Written consents from all participants involved in this study were obtained.

## Author Contributions

MP designed the research, conducted the data analyses, and wrote the first draft, and reviewed and approved the final manuscript. CN designed the research, supervised the data analyses, and reviewed and approved the final manuscript.

## Conflict of Interest Statement

The authors declare that the research was conducted in the absence of any commercial or financial relationships that could be construed as a potential conflict of interest.
